# Integrated Genomic Profiling of Pediatric Acute Lymphoblastic Leukemia: Genomic Landscape, Risk Stratification and Association of RAS Pathway Mutations with Early Treatment Response

**DOI:** 10.3390/ijms27104517

**Published:** 2026-05-18

**Authors:** Andreea Stefan-Hodorogea, Letitia Radu, Andra Marcu, Andreea Serbanica, Ana Bica, Cristina Jercan, Ana Marcu, Dumitru Jardan, Cerasela Jardan, Onda Calugaru, Mihaela Dragomir, Codruta Popa, Anca Gheorghe, Karina Vihta, Simona Dima, Anca Colita

**Affiliations:** 1 Faculty of General Medicine, “Carol Davila” University of Medicine and Pharmacy, 020022 Bucharest, Romania; andreea-iulia.stefan@drd.umfcd.ro (A.S.-H.); andreea.serbanica@umfcd.ro (A.S.); ana-maria.birsan@drd.umfcd.ro (A.B.); cristina.jercan@umfcd.ro (C.J.); ana-maria.marcu@umfcd.ro (A.M.); cerasela.jardan@umfcd.ro (C.J.); onda-tabita.lupu@drd.umfcd.ro (O.C.); delia.popa@umfcd.ro (C.P.); simona.dima@umfcd.ro (S.D.); anca.colita@umfcd.ro (A.C.); 2 Department of Pediatrics, Fundeni Clinical Institute, 022328 Bucharest, Romania; 3 Molecular Biology Laboratory, Medlife Clinic, Calea Grivitei No. 371, 012244 Bucharest, Romania; djardan@medlife.ro (D.J.);; 4 Genetics Laboratory, Fundeni Clinical Institute, 022328 Bucharest, Romania; dragomir_mihaela28@yahoo.com; 5 Flow Cytometry Laboratory, Fundeni Clinical Institute, 022328 Bucharest, Romania; ancagheorghe15@gmail.com; 6 Biostatistics, Fundeni Clinical Institute, 022328 Bucharest, Romania; 7 Center of Excellence in Translational Medicine (CEMT), Fundeni Clinical Institute, 022328 Bucharest, Romania

**Keywords:** acute lymphoblastic leukemia, next-generation sequencing, RAS pathway mutations

## Abstract

Acute lymphoblastic leukemia (ALL) is the most common pediatric malignancy and genomic profiling has become increasingly vital for risk stratification. We conducted a retrospective single-center study including 96 newly diagnosed pediatric patients with ALL and mixed phenotype acute leukemia to characterize the genomic landscape using conventional and molecular cytogenetics, multiplex ligation-dependent probe amplification and targeted next-generation sequencing and to evaluate associations with treatment response and survival. Mutation co-occurrence analysis revealed a NRAS/KRAS/JAK cluster and an *ETV6*::*RUNX1* cluster characterized by relative mutational exclusivity, with additional associations between *NOTCH1*–*CDK4* and *CDK4*–CCND3 alterations. Del*CDKN2A/CDKN2B* alterations showed a trend toward higher day-15 minimal residual disease (MRD) levels and inferior 2-year event free survival rate (EFS). Del*IKZF1*-positive cases showed lower 2-year EFS. In contrast, *ETV6*::*RUNX1* and high hyperdiploidy were associated with favorable early response and EFS. Del*PAX5* did not independently influence outcome. *ZNF384* positive cases showed higher early MRD levels despite excellent survival outcomes. NRAS/KRAS mutations were significantly associated with higher positive day-15 MRD (Wilcoxon, *p* = 0.0067) and remained independently associated after adjustment for white blood cell count and cytogenetic subgroup. Intermediate risk (IR) and high-risk groups showed comparable 2-year EFS, indicating limited discrimination by conventional risk stratification. The IR group displayed a heterogeneous genomic profile, with NRAS/KRAS, Ph-like mutations and del*CDKN2A/CDKN2B* among the most frequent alterations. These observations highlight the potential of integrated genomic profiling to refine risk stratification, particularly by identifying clinically relevant subgroups within the IR category.

## 1. Introduction

Acute lymphoblastic leukemia (ALL) accounts for approximately 25% of all pediatric cancers and 75–80% of all childhood leukemias, making it the most common childhood malignancy. The incidence of ALL is estimated at 3–4 cases per 100,000 children per year, with a peak occurrence between 2 and 5 years of age [1,2,3]. Over the past decades, survival has improved significantly, with 5-year event-free survival rates (EFS) now exceeding 85% in developed countries. Much of this progress has come from risk-adapted therapy and improved stratification methods, supported by better characterization of leukemic cells. However, despite this progress, relapse remains a challenge and is the leading cause of ALL-related mortality [1,2].

Relapsed ALL is associated with poor outcomes, with survival rates dropping to 30–50%, depending on factors such as the timing of relapse and phenotypic features of the leukemic clone. Approximately 20% of pediatric patients experience relapse, often with a more aggressive chemoresistant dominant clone. Progress in molecular characterization of leukemic clones has significantly improved risk assessment, moving beyond traditional morphological and immunophenotypic classification. In recent years, numerous studies have focused on high-resolution profiling, which has led to increased interest in copy number alterations (CNAs) and point mutations. These alterations have provided valuable insights into leukemogenesis and risk assessment [4,5,6].

The growing importance of genomic profiling is also reflected in the 2022 World Health Organization (WHO) classification of hematolymphoid malignancies, which places greater emphasis on genetically defined disease entities. Newly recognized subtypes include B-cell ALL with *DUX4, MEF2D, ZNF384, NUTM1, MYC* rearrangements, and *PAX5* alterations as well as *ETV6*::*RUNX1*-like and *TCF3::HLF* leukemias [7].

Recurrent Deletions affecting genes involved in lymphoid differentiation and cell-cycle regulation, like *IKZF1*, *CDKN2A/CDKN2B* and *PAX5*, are of particular clinical relevance. *IKZF1* Deletions as well as the *IKZF1*plus profile have been associated with treatment resistance and inferior prognosis and are introduced into current risk stratification systems [8,9,10]. Del*CDKN2A/CDKN2B* have also been linked to adverse clinical features and poorer outcome in pediatric ALL [11,12,13]. By contrast, the prognostic role of *PAX5* alterations appears to depend in part on their co-occurrence with other lesions, while germline *PAX5* variants further support the biological relevance of this gene in leukemogenesis and cancer development [14,15,16,17,18].

Additional genomic subgroups further illustrate the heterogeneity of pediatric ALL. Intrachromosomal amplification of chromosome 21 (iAMP21) is a rare but clinically relevant abnormality associated with poor treatment response and exclusion from standard-risk classification [19]. PhilaDelphia chromosome-like (Ph-like) ALL represents a high-risk subgroup characterized by kinase-activating alterations involving cytokine receptor and signaling pathways, most commonly ABL-class and JAK-STAT lesions [20,21,22]. These alterations are of particular interest because they may be therapeutically targetable. Early experiences reported by St. Jude Children’s Research Hospital support the integration of next-generation sequencing into the identification of lesions with potential therapeutic relevance and targeted therapy in the induction treatment [23,24]. Alterations affecting the RAS pathway are among the most common genetic alterations, not only in ALL, but in all types of cancer and have been implicated in clonal evolution and treatment resistance [25,26]. Rearrangements involving *ZNF384* define a distinct molecular subgroup of B-cell ALL, frequently associated with atypical immunophenotypic features and variable treatment response [27,28].

Advances in genomic technologies now allow broader characterization of leukemic genomes. Conventional cytogenetics remains central for detecting large chromosomal abnormalities, while multiplex ligation-dependent probe amplification (MLPA) provides a rapid and cost-effective approach for recurrent CNAs [29,30,31]. Targeted next-generation sequencing (NGS) enables simultaneous detection of multiple somatic alterations across clinically relevant pathways and offers an integrated view of leukemia biology with potential diagnostic, prognostic and therapeutic implications [32,33,34].

The primary objective of this study was to evaluate the association between recurrent genomic alterations and clinical outcomes in newly diagnosed pediatric ALL and mixed phenotype acute leukemia (MPAL), focusing on early treatment response assessed by minimal residual disease (MRD), survival analysis, namely EFS and overall survival (OS), and relationship with protocol-defined risk stratification and corresponding treatment allocation, using an integrated genomic approach.

## 2. Results

A total of 96 pediatric patients diagnosed with ALL were included in the study. Baseline demographic, clinical and immunophenotypic characteristics of the cohort are summarized in Table 1. According to the protocol stratification criteria, 13 cases were treated on standard risk arm, 56 on the intermediate risk arm and 27 on the high-risk arm.

Treatment response assessed by FC-MRD at days 15, 33 and before consolidation (day 78) is summarized in Table 2.

Overall survival outcomes were favorable in the cohort. The estimated 2-year EFS and OS were 87.5% (95% CI: 76–93) and 90.6% (95% CI: 82–95), respectively, with a median follow-up of 18 months (IQR, 9–24). The number of patients at risk at 24 months was 38. The relapse rate was 7.1%; all events occurred early (≥18 months after primary diagnosis and <6 months after the completion of primary therapy) or very early (<18 months after primary diagnosis). Two-year EFS was 85.2% (95% CI: 67–98) in the HR group, 85.7% (95% CI: 73–94) in the IR group and 100% (95% CI: 75–100) in the SR group, with comparable outcomes between the HR and IR groups.

### 2.1. Patient Cohort and Overall Treatment Response

Conventional cytogenetics showed a predominance of normal karyotype (28%), followed by high hyperdiploidy (HeH) (17%) and complex karyotype (12%), with other abnormalities occurring at low frequencies (Table 3).

Most cases were FISH-negative (89%), with *KMT2A* and *TCF3* rearrangements each detected in 3% of patients, while other abnormalities were rare. Overall, fusion transcripts were identified in 22% of cases, predominantly *ETV6::RUNX1* (17%), followed by *KMT2A*-rearranged fusions (4%), with other alterations occurring at low frequencies (Table 3).

**Figure 1:**
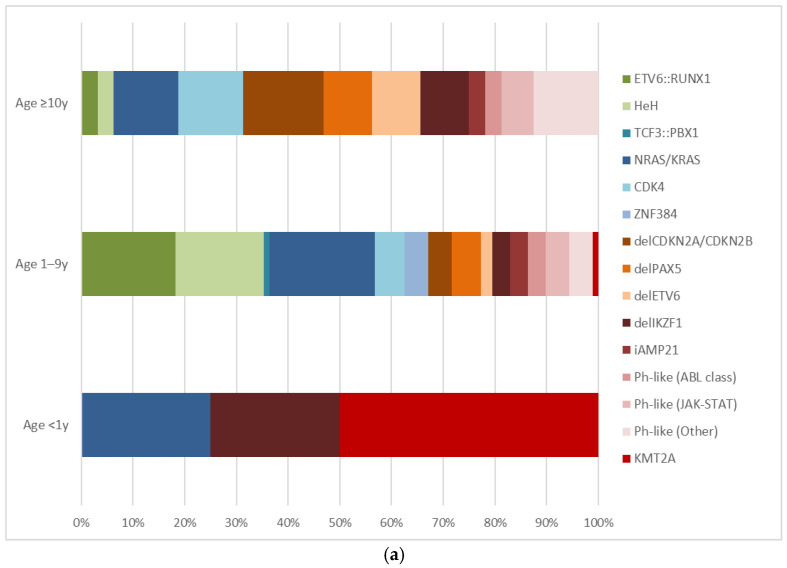

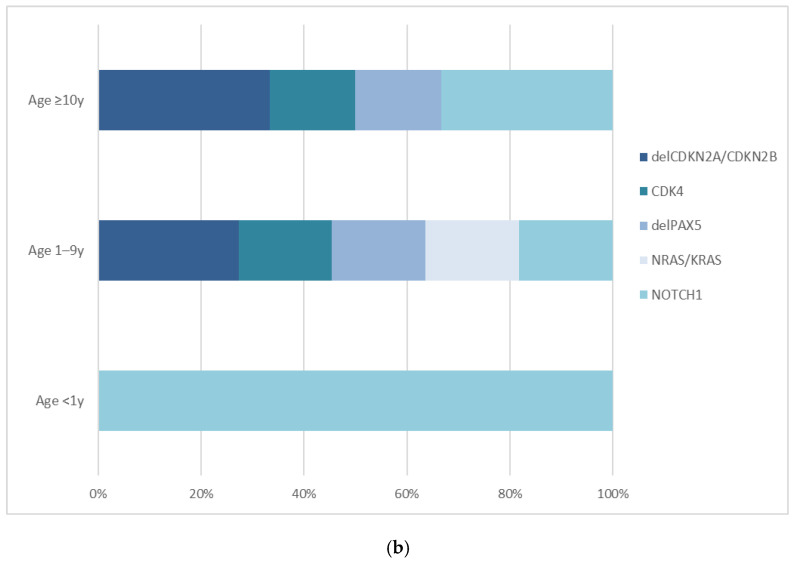
Distribution of genetic alterations across age categories in B-cell acute lymphoblastic leukemia (**a**) and T-cell acute lymphoblastic leukemia (**b**). Colors indicate prognostic relevance according to current risk stratification protocols: green, standard-risk markers; blue, not yet classified; red/orange, generally associated with adverse prognostic features. Each bar represents the absolute number of patients within the indicated age band carrying the corresponding alteration; patients harboring more than one alteration are counted in each relevant category. Abbreviations: HeH, high hyperdiploidy; iAMP21, intrachromosomal amplification of chromosome 21.

Figure 1a,b illustrates the distribution of the main cytogenetic and molecular alterations across age categories in B-cell and T-cell ALL.

Detailed treatment response and survival outcomes according to genetic subtypes are presented in Table 4.

### 2.2. Copy Number Alterations and Association with Early Treatment Response and Survival

CNAs were assessed by MLPA in 96 patients. The most frequent CNAs were Del*CDKN2A/CDKN2B*, followed by Del*PAX5* and Del*IKZF1*, with iAMP21 identified in a small number of cases (4%). Del*IKZF1* and iAMP21 were observed exclusively in B-cell ALL, while Del*CDKN2A/CDKN2B* occurred in both B- and T-cell ALL, most frequently in B-common ALL (6/13), and Del*PAX5* predominantly in B-cell ALL (8/11). FC-MRD evaluations and survival outcomes are summarized in Table 4.

**Figure 2:**
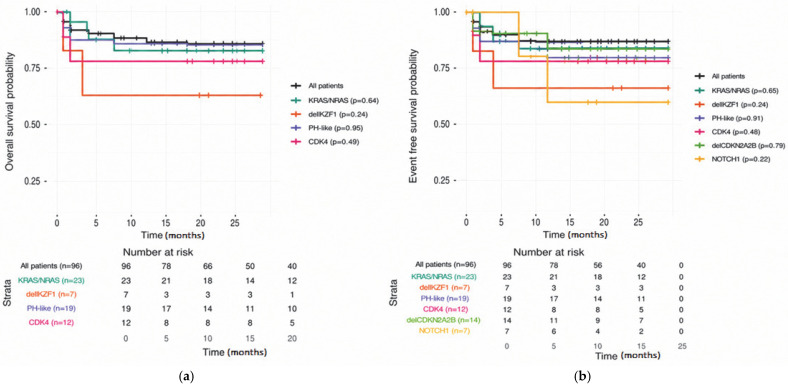
Two-year EFS (**a**) and OS (**b**) according to selected genomic alterations in pediatric acute lymphoblastic leukemia. Kaplan–Meier curves showing only alterations with at least two events (relapse/death) were included in the analysis; time on the *x*-axis is expressed in months from diagnosis. Log-rank *p*-values are indicated for each comparison. No comparison reached statistical significance. The number of patients at risk for the main timepoints is provided in the table below the curve.

Del*IKZF1* was associated with inferior survival outcomes. Among del*IKZF1*-positive patients (n = 7), early FC-MRD response was heterogeneous and one early medullary relapse was observed. Two-year EFS and OS were both 71.4% (95% CI: 38–100), lower than in the overall cohort (Figure 2). Five cases fulfilled the criteria for an *IKZF1*plus profile, most frequently in association with del*CDKN2A/CDKN2B*. The *IKZF1*plus subtype was defined as del*IKZF1* co-occurring with del*CDKN2A/CDKN2B*/*PAX5*/PAR1 (P2RY8-CRLF2) in the absence of ERG Deletion.

Del*CDKN2A/CDKN2B* and del*PAX5* were associated with variable early FC-MRD response but showed outcomes comparable to the overall cohort (Figure 2). Within the del*CDKN2A/CDKN2B* positive subgroup, FC-MRD at day 15 was heterogeneous, with a trend toward inferior response (Table 4). By day 33, only two patients remained FC-MRD positive, and by day 78, a single patient had detectable FC-MRD. One patient developed an early CNS relapse. Most del*PAX5* positive patients achieved FC-MRD negativity during induction therapy. The only case with persistent MRD during follow-up also carried del*CDKN2A/CDKN2B* and subsequently developed an early CNS relapse (Figure 2).

Patients positive for *iAMP21* alteration showed favorable response and outcomes. All patients achieved FC-MRD negativity after induction therapy and no relapse events were recorded during follow-up (Figure 2).

Additional CNAs were rare and often co-occurred with other alterations. These included del*ETV6* (n = 5), del*EBF1* (n = 2) and del*RB1* (n = 2). *ETV6* deletions frequently co-occurred with *CDKN2A/CDKN2B* and *PAX5* deletions, while all *EBF1* deletions occurred concomitantly with *ETV6* deletions and were identified in high-risk patients. During follow-up, one patient with combined del*RB1* and del*CDKN2A/CDKN2B* developed an isolated CNS relapse.

### 2.3. Targeted NGS Findings: Mutational Landscape, Co-Occurrence and Associations with Treatment Response and Outcome

Targeted NGS analysis was successfully performed in 89 samples; the remaining samples could not be analyzed due to insufficient DNA quality or quantity and technical issues during library preparation. The most frequent alterations identified were RAS pathway mutations, followed by *ETV6*::*RUNX1*, *NOTCH1*, *ZNF384* rearrangements, *CDK4*, FLT3 and *PAX5* alterations.

**Figure 3:**
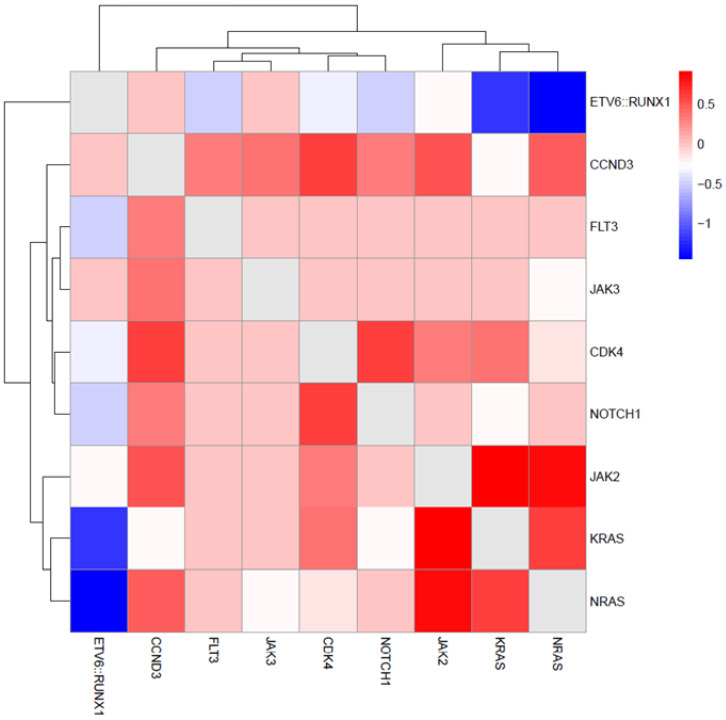
Mutational co-occurrence patterns. Heatmap of signed log-transformed *p*-values for pairwise associations between recurrent gene alterations detected by targeted NGS in the cohort (≥0.05 frequency).

Co-occurrence analysis of recurrent mutations (Figure 3) revealed three principal mutational groups: a separate *ETV6*::*RUNX1* cluster, a signaling cluster including NRAS, KRAS and JAK2 and a third cluster encompassing the remaining recurrent alterations. A positive association was observed between RAS pathway alterations and JAK mutations, with the strongest association observed between JAK2 and NRAS (*p* = 0.03, OR = 8.4). Mutual exclusivity was noted between *KRAS/NRAS* and *ETV6*::*RUNX1* (*p* = 0.03, OR = 0). Additional numerical clustering was observed between *NOTCH1* and *CDK4* (*p* = 0.2, OR = 2.7) and between *CDK4* and CCND3 (*p* = 0.2, OR = 2.7).

**Figure 4:**
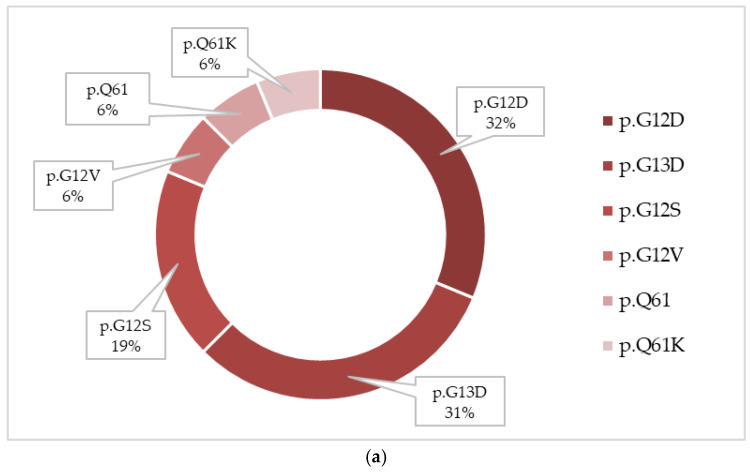

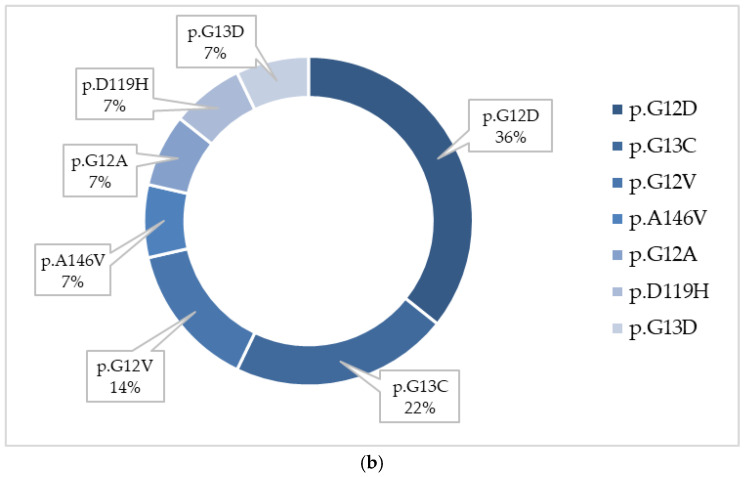
Distribution of NRAS (**a**) and KRAS (**b**) mutation variants in the cohort. The frequency of each variant is expressed as a percentage of total mutated cases for each gene.

**Figure 5:**
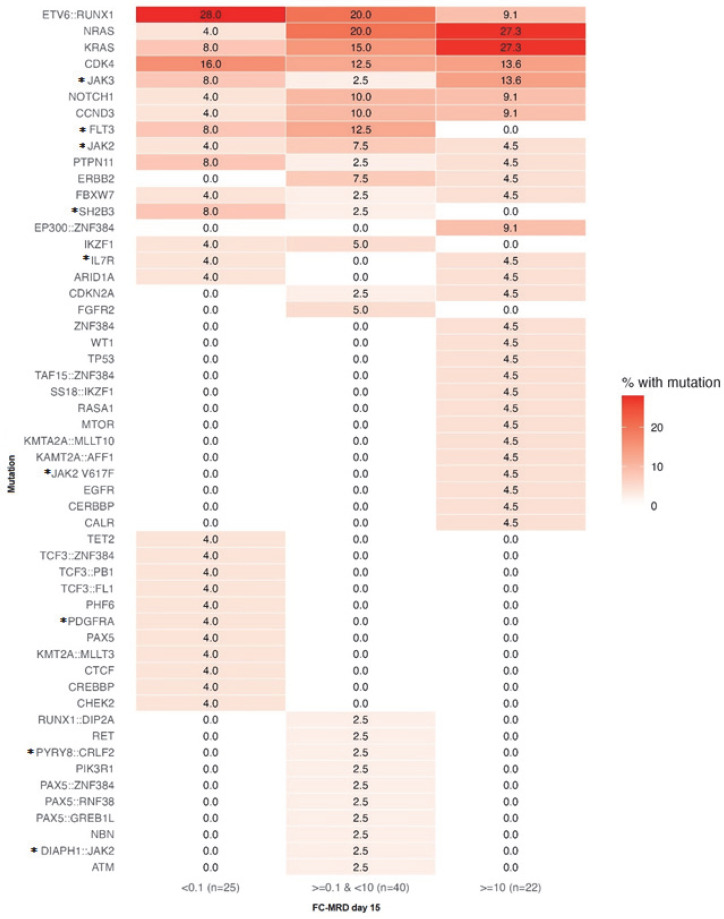
Distribution of genomic alterations across day-15 minimal residual disease categories (FC-MRD). Heatmap showing the percentage of patients with each mutation within each day-15 FC-MRD category. Note that individual patients may harbor more than one mutation (mutation categories are not mutually exclusive); therefore, column totals may exceed 100%. Ph-like-associated alterations are marked with an asterisk (*) and are defined in the Section 4.

*KRAS/NRAS* mutations, identified in 23 cases, were significantly associated with higher day-15 FC-MRD levels (Wilcoxon rank-sum, *p* = 0.0067). NRAS mutations mainly involved codons 12 and 13, with p.G12D as the most frequent variant, while KRAS mutations were predominantly represented by p.G13D alterations (Figure 4). These alterations were most frequent in the ≥10% FC-MRD category, where they were identified in approximately 50% of patients, compared with around 10% in the <0.1% subgroup (Figure 5). This association was further evaluated in multivariable analyses (Section 2.5). One patient presented with very high day-15 FC-MRD (52%) without other detectable mutations, with persistence of FC-MRD positivity at day 33. Survival outcomes were comparable to the overall cohort. Two-year OS and EFS were 88.5% (95% CI: 76–100) and 84.6% (95% CI: 71–98), respectively, with two relapse events observed in this subgroup (Table 4).

*ETV6::RUNX1* was associated with favorable early FC-MRD clearance and superior survival outcomes. The fusion was identified in 17 patients, all with B-common ALL and predominantly aged 1–10 years. At day 15, 28% of cases had MRD <0.1% (Figure 5), and all evaluable patients achieved FC-MRD negativity by day 33 (*p* = 0.057). This subgroup demonstrated favorable survival. Two-year OS and EFS were both 94.1% (95% CI: 83–100), higher than in negative cases.

**Figure 6:**
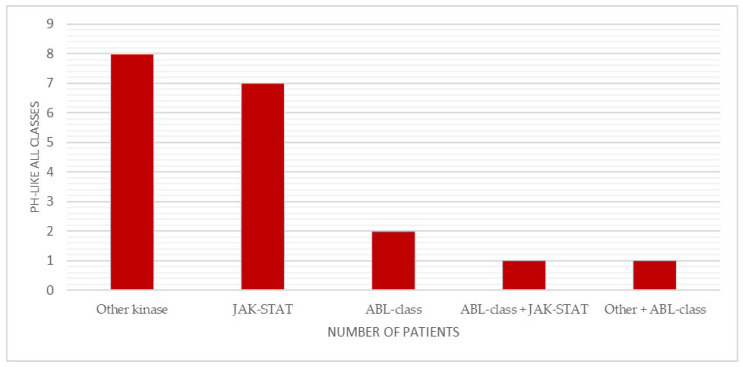
Distribution of each Ph-like acute lymphoblastic leukemia class in the cohort. Bar chart showing the number of patients with alterations involving JAK–STAT signaling, ABL-class fusions, other kinase pathways and combined alterations.

*Ph-like* alterations (as defined in the Section 4) were associated with intermediate FC-MRD response and slightly lower survival compared with negative cases. These alterations were identified in 19 patients (19%), predominantly in B-common ALL and more frequently in older male individuals. The distribution of Ph-like classes is shown in Figure 6. At day 15, most Ph-like-positive cases showed FC-MRD levels < 10%, with only a few patients remaining FC-MRD positive at day 33 (Figure 5). Two-year OS and EFS were 89.5% (95% CI: 76–100) and 84.2% (95% CI: 68–100), respectively.

*ZNF384* alterations were associated with higher early FC-MRD levels but favorable survival outcomes. These alterations were identified in 6 patients, mainly aged 1–10 years and predominantly in B-common ALL and MPAL cases. Most involved gene fusions, with EP300 as the most frequent partner, alongside *PAX5*, TCF3 and TAF15 and one intragenic mutation. At day 15, most cases showed high FC-MRD levels, suggesting a poorer early response, followed by Delayed FC-MRD clearance, with low-level persistence at day 33 (2/6 patients) (Figure 5). Despite this, no relapse or death events were observed, with a 2-year OS and EFS of 100% (95% CI: 60–100).

*NOTCH1* mutations were associated with heterogeneous early FC-MRD response and lower EFS, without an apparent impact on OS. *NOTCH1* mutations (n = 7), associated with T-cell ALL, showed a broad age distribution. Treatment response at day 15 was heterogeneous (Figure 5). Two relapse events (medullary and CNS) were recorded, resulting in a 2-year OS of 100% (95% CI: 60–100) and a 2-year EFS of 71.4% (95% CI: 38–100). *CDK4* alterations showed no impact on treatment response or survival outcomes. These were identified in 12 patients, predominantly in B-cell ALL, particularly common B-cell ALL.

### 2.4. Multivariable Analysis of Genomic Alterations and Clinical Outcomes

Multivariable Cox proportional hazards regression analysis for 2-year EFS was performed adjusting for cytogenetic subtype, day-15 FC-MRD category and WBC at diagnosis. *KRAS/NRAS* alterations did not have independent statistical significance after adjustment (adjusted HR 2.05, 95% CI: 0.52–8.11; *p* = 0.309). Similarly, del*IKZF1* (adjusted HR 1.49, 95% CI: 0.16–13.68; *p* = 0.726) and del*CDKN2A/CDKN2B* (adjusted HR 0.62, 95% CI: 0.07–5.76; *p* = 0.676) were not independent predictors of EFS.

**Figure 7:**
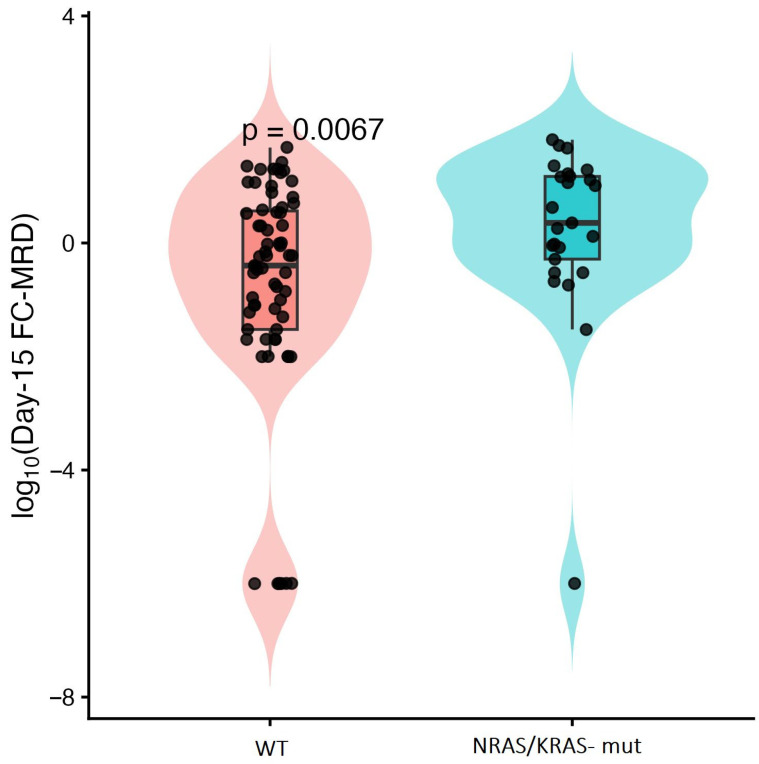
Log10-transformed day-15 FC-MRD levels according to *KRAS/NRAS* mutational status (Wilcoxon rank-sum test). Violin plots with overlaid boxplots and individual patient values (black dots) illustrating log10-transformed day-15 FC-MRD levels in *KRAS/NRAS*-mutated and wild-type (WT) cases. *KRAS/NRAS*-mutated patients demonstrated significantly higher FC-MRD levels compared with WT patients (Wilcoxon rank-sum test, *p* = 0.0067). The central box represents the interquartile range and the horizontal line indicates the median value.

In linear regression models of log10-transformed day-15 FC-MRD values, *KRAS/NRAS* mutations were associated with significantly higher MRD levels in both univariate and adjusted analyses (Figure 7). After adjustment for WBC count and cytogenetic subgroup, *KRAS/NRAS* mutations remained independently associated with higher FC-MRD levels (adjusted β = 1.13, *p* = 0.020), corresponding to approximately 13-fold higher day-15 FC-MRD values in mutated cases vs. wildtype.

### 2.5. Survival Outcomes According to Protocol-Defined Risk Groups

Genomic alterations showed distinct distributions across protocol-defined risk groups. *ETV6*::*RUNX1* and HeH were predominantly observed in the SR subgroup. The IR subgroup demonstrated substantial genomic heterogeneity, with recurrent *KRAS/NRAS* (30%), Ph-like (23%), del*CDKN2A/CDKN2B* (18%) and *PAX5* (16%) alterations representing some of the most frequent findings. A more detailed overview of the genomic distribution within the IR subgroup is provided in Supplementary Figure S1. In the HR subgroup, *KRAS/NRAS* mutations predominated (41%), with *CDKN2A/CDKN2B* and *IKZF1* deletions also frequently observed.

**Figure 8:**
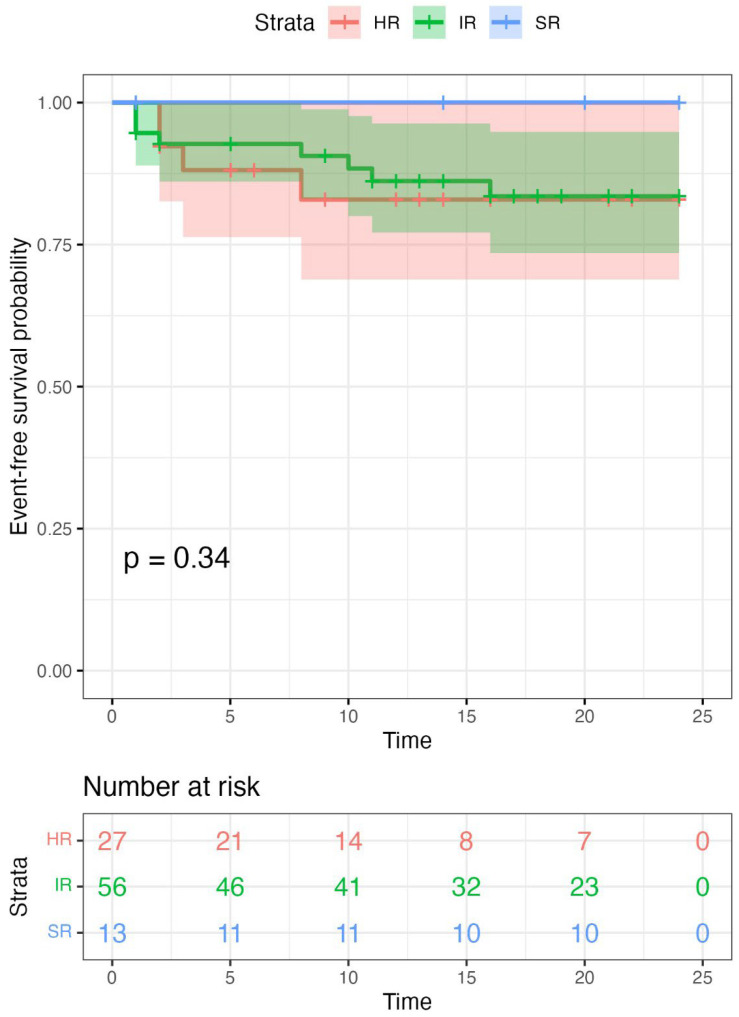
Two-year event-free survival of patients treated according to protocol-defined risk groups. Kaplan–Meier curves illustrating 2-year EFS between risk groups. Shaded areas represent 95% confidence intervals. Tick marks indicate censored observations. Log-rank *p* = 0.34. Risk groups (SR, standard-risk; IR, intermediate-risk; HR, high-risk) were defined by the ALLIC BFM 2022 protocol as detailed in Section 4.2. Numbers of patients at risk at main timepoints are listed beneath the curves for each arm.

Survival outcomes were comparable between IR and HR subgroups. The SR subgroup showed consistently high survival, with no events recorded during follow-up. Two-year EFS was 85.7% (95% CI: 73–94) in the IR subgroup and 85.2% (95% CI: 67–98) in the HR subgroup (log-rank *p* = 0.34), indicating similar survival patterns between these groups (Figure 8).

## 3. Discussion

CNAs represented a major component of genomic abnormalities in our cohort. Del*CDKN2A/CDKN2B* were the most frequent CNAs and were more commonly observed in patients with a normal karyotype background. *CDKN2A/CDKN2B* deletions have been associated with increased proliferative signaling and adverse clinical features in multiple pediatric studies [12,13]. In our cohort, del*CDKN2A/CDKN2B* positive cases had a less favorable early treatment response. These observations suggest clinically relevant suboptimal early treatment response in this subgroup, consistent with the findings reported by Sarangarajan et al. [35] in a large meta-analysis of 2532 patients, which demonstrated an increased relapse risk and inferior survival outcomes in children with this alteration. Del*IKZF1* is incorporated into current risk stratification systems and frequently associated with high-risk disease [8,9,10,11]. Patients with del*IKZF1* in our cohort showed lower survival rates compared with those without the alteration (71.4% [95% CI: 38–100] vs. 88.6% [95% CI: 82–95]). Although statistical significance was not reached given the limited number of del*IKZF1*-positive cases, the >20% absolute difference in survival is clinically relevant. Similar observations were reported by Felice et al. [36] in a large cohort of more than 1000 pediatric patients treated with BFM based protocols, where del*IKZF1* was associated with significantly inferior outcomes. Our findings therefore support the prognostic relevance of *IKZF1* alterations.

Jung et al. [37] demonstrated inferior OS in pediatric ALL with *PAX5* mutations, which frequently co-occurred with deletions of *IKZF1* and *CDKN2A/CDKN2B*. Similarly, in our cohort, del*PAX5* alone did not adversely impact survival, while the patient with additional co-occurring CNAs developed an early CNS relapse. In contrast, iAMP21 was not associated with inferior OS or EFS in our cohort. Given the small number of cases, the power to detect outcome differences was limited and the adverse impact traditionally attributed to this lesion may be attenuated by contemporary risk-adapted therapy.

In our study, pairwise mutational association analysis revealed a distinct signaling-related cluster comprising *KRAS/NRAS* and JAK. In large genomic analyses of Ph-like ALL, RAS pathway mutations have similarly been reported within the spectrum of kinase-activating alterations alongside JAK alterations, supporting the biological convergence of these cases toward activated cytokine receptor and proliferative signaling pathways [38]. RAS pathway mutations were among the most frequent alterations. In addition, the majority of these mutations involved hotspot variants affecting codons 12 and 13, which represent the most common mutational sites in pediatric ALL and contribute to MAPK pathway activation [39]. These alterations were statistically significantly associated with higher positive day-15 FC-MRD levels and remained significant after adjustment for WBC count and cytogenetic subgroup in multivariable linear regression analysis. Activating mutations in NRAS and KRAS are known to promote constitutive proliferative signaling and relative chemotherapy resistance [26]. The association between RAS mutations and higher day-15 FC-MRD levels in our cohort supports their role in chemoresistance. This observation is consistent with the data reported by Jerchel et al. [40], who analyzed patients treated within the MRD-stratified Dutch Childhood Oncology Group ALL10 (DCOG ALL10) protocol and demonstrated that clonal RAS pathway mutations were associated with early treatment response. Moreover, RAS-mutant leukemic cells exhibited increased resistance to glucocorticoids and vincristine ex vivo, providing a biological explanation for Delayed MRD clearance. Although targeted RAS inhibitors are not yet established in pediatric ALL, emerging data from solid tumors and preclinical studies suggest that these alterations may represent potential therapeutic targets [39].

*ETV6*::*RUNX1*-positive cases demonstrated favorable early FC-MRD clearance and superior survival outcomes, consistent with its established good prognosis profile in pediatric ALL [41]. In our cohort, *ETV6*::*RUNX1* genetic subtype formed a distinct cluster, with no additional recurrent mutations detected by NGS. *ETV6*::*RUNX1* and HeH were associated with consistently favorable outcomes.

The frequency of Ph-like ALL was comparable to that reported in other studies. Ph-like-positive cases in our cohort showed a tendency toward inferior early FC-MRD response, consistent with the adverse prognosis described in previous reports [21,22,23]. In particular, Chiaretti et al. [42] reported that Ph-like ALL is associated with MRD persistence and poor outcome. In our cohort, Ph-like-positive patients also showed slightly lower 2-year OS and EFS compared with Ph-like-negative.

*NOTCH1* alterations showed clustering with *CDK4* (OR > 1), consistent with the known role of *NOTCH1* signaling in driving leukemic proliferation via direct transcriptional activation of *CDK4*/*6* and promotion of G1/S cell-cycle progression [43]. They showed lower EFS despite preserved OS in our cohort, reflecting the occurrence of relapse events without impact on mortality. This finding is consistent with the recent meta-analysis by Al Kuwari et al. [44], which included 11 studies (2039 patients) and demonstrated improved EFS in *NOTCH1* positive T-cell-ALL, with a trend toward superior OS.

Among our patients, B/myeloid MPAL was associated with *ZNF384* fusions, consistent with other studies [45,46]. The *ZNF384*-positive subtype was associated with higher early FC-MRD levels, but favorable EFS in our cohort, suggesting delayed MRD clearance with preserved long-term sensitivity to therapy. Previous studies have reported variable early response to treatment in *ZNF384*-rearranged ALL, depending on the partner gene [27,28]. *CDK4* and CCND3 alterations showed a co-occurrence pattern (OR > 1), consistent with the established functional interaction, in which CCND3 has been shown to represent the dominant D-type cyclin essential for leukemic cell maintenance [47].

Our analysis revealed considerable genomic heterogeneity within the IR subgroup, with RAS pathway mutations, *CDKN2A/CDKN2B* and *PAX5* deletions among the most frequent alterations. Despite being treated within the same protocol-defined risk category, IR patients demonstrated outcomes comparable to the HR group, suggesting limited discrimination by conventional risk stratification. These findings raise the possibility that a subset of IR patients may correspond to a higher-risk biological subgroup and could potentially benefit from treatment intensification. In particular, RAS pathway mutations may contribute to defining such a subgroup, although this hypothesis requires validation in larger cohorts before clinical implementation.

Targeted genomic profiling may identify clinically relevant subgroups within the conventional risk categories, particularly among intermediate-risk patients, supporting the need for refined molecular risk stratification, to enable more precise treatment modulation.

The limitations of this study are the relatively small cohort size and short follow-up duration, restricting the ability to draw definitive conclusions regarding long-term relapse risk. These estimates should be interpreted with caution given the limited number of patients reaching the 2-year landmark. Extended observation and validation in larger cohorts are warranted to confirm the prognostic implications of these genomic findings. To our knowledge, this is the most comprehensive integrated genomic analysis of pediatric ALL conducted in Romania to date and, given the national referral profile of our institution, the cohort may reflect the molecular landscape of the disease in our region.

## 4. Materials and Methods

### 4.1. Study Design and Data

This is a retrospective observational study conducted between December 2022 and August 2025 at the Fundeni Clinical Institute, Department of Pediatrics, Bucharest, Romania. The study included pediatric and adolescent patients aged 0–18 years newly diagnosed with ALL or MPAL. Inclusion criteria were: (i) age 0–18 years at diagnosis; (ii) confirmed diagnosis of ALL or MPAL according to the 2017 WHO Classification of Tumors of Haematopoietic and Lymphoid Tissues; (iii) treatment initiation according to the ALLIC BFM 2022 protocol. Exclusion criteria were: (i) prior cytotoxic chemotherapy or corticosteroid exposure before bone marrow analysis; (ii) relapsed or refractory disease at inclusion; (iii) secondary leukemia following another malignancy and (iv) absence of informed consent. Clinical and laboratory data were recorded, including age, sex, white blood cell count, immunophenotype, cytogenetic and molecular findings and treatment response (FC-MRD status) at days 15, 33 and 78, as defined by protocol. All patients received frontline therapy according to the same ALLIC BFM 2022 backbone protocol, with treatment intensity adjusted only according to protocol-defined risk groups (SR, IR and HR), as detailed in Section 4.2. This study was conducted and reported in accordance with the STROBE (Strengthening the Reporting of Observational Studies in Epidemiology) guidelines.

### 4.2. Diagnosis, Molecular Profiling, MRD and Protocol

The diagnosis was established based on the evaluation of bone marrow aspirate samples. Immunophenotypic analysis was performed by flow cytometry (FC) using the BD FACSLyric™ system (Becton Dickinson, Franklin Lakes, NJ, USA) and data were interpreted with Infinicyt™ version 2.0 (Cytognos, Salamanca, Spain). The classification of leukemic cells was carried out according to the criteria outlined in the 2017 WHO Classification of Tumors of Haematopoietic and Lymphoid Tissues. Conventional cytogenetic analysis with GTG banding and molecular with fluorescence in situ hybridization (FISH) was performed according to standard protocols. Recurrent ALL-associated fusion transcripts were assessed by multiplex RT-PCR. CNAs were analyzed using MLPA (P335 and P327 iAMP21-ERG kits) and targeted mutational profiling was performed using the Illumina TruSight™ Oncology 500 platform (Illumina, San Diego, CA, USA). Detailed methodological procedures are provided in the supplementary methods (Appendix A).

Ph-like ALL was defined by the absence of BCR::ABL1 fusion in B-cell ALL and the presence of kinase-activating including ABL-class rearrangements (ABL1, ABL2, PDGFRA, PDGFRB, FGFR1), JAK-STAT rearrangements and/or mutations (CRLF2, EPOR, JAK1, JAK2, JAK3, TYK2, SH2B3, IL7R) and other rearrangements in FLT3, NTRK3, LYN and PTK2B genes.

The *IKZF1*plus subtype was defined as del*IKZF1* co-occurring with del*CDKN2A/CDKN2B*/*PAX5*/PAR1 (P2RY8-CRLF2) in the absence of ERG deletion.

MRD was evaluated by multiparametric FC on bone marrow aspirate samples using panels of leukemia-associated immunophenotypes tailored to each patient’s diagnostic profile. A minimum of 100.000 nucleated events were acquired per sample and analyzed using Kaluza^®^ software version 2.1 (Beckman Coulter, Brea, CA, USA), corresponding to a sensitivity of 10^−4^. MRD was assessed at protocol-defined timepoints: day 15, day 33 (end of induction) and day 78 (end IB/before consolidation).

Risk stratification under the ALLIC BFM 2022 protocol is based on a combination of clinical features at diagnosis (immunophenotype and genetic features), prednisone response (for T-cell ALL) and MRD response at days 15, 33 and 78. Patients are allocated to SR, IR or HR treatment arms, with progressive treatment intensification. Favorable genetic subtypes (*ETV6*::*RUNX1* and HeH, iAMP21 negative) with <0.1% FC-MRD on day 15 are associated with SR assignment and receive a less intensive treatment. HR genetic features include BCR::ABL1, KMT2A rearrangements, hypodiploidy, TCF3::HLF, iAMP21 and *IKZF1*plus. FC-MRD ≥ 10% at day 15 or persistent positivity (≥0.05%) at later time points (day 33 or 78) leads to HR assignment. Treatment follows the BFM backbone [48].

### 4.3. Statistical Analysis

The outcomes evaluated included EFS, defined as the time from diagnosis to relapse, death or last follow-up, and OS, defined as the time from diagnosis to death. Vital status was verified for all the patients through the national health insurance database, allowing inclusion of patients lost to institutional follow-up.

All statistical analyses were performed using IBM^®^ SPSS (version 26) and the R 4.5.2 program. Survival analyses for EFS and OS were conducted using the Kaplan–Meier method and differences between groups were assessed with the log-rank test. Linear regression models were fitted to evaluate independent association between KRAS/NRAS alterations and early treatment response after adjustment for WBC count and cytogenetic features. Differences in continuous log10-transformed day-15 FC-MRD values between groups were assessed using the Wilcoxon rank-sum test. Multivariable Cox proportional hazards regression analysis was performed to evaluate the independent association of *KRAS/NRAS* alterations, del*IKZF1* and del*CDKN2A/CDKN2B* with 2-year EFS after adjustment for WBC count and cytogenetic subgroup. Associations between categorical variables were analyzed using the Chi-square test. When more than 25% of the expected cell counts were below 5, Fisher’s exact test was applied. Statistical dependence between recurrent gene alterations (>5%) was evaluated using Fisher’s exact test (mutual exclusivity test) and visualized as heatmaps of odds ratios and *p*-values. A *p*-value of less than 0.05 was considered statistically significant.

The authors used an AI-based tool for language editing and spelling correction. No AI tools were used for data analysis, interpretation or generation of scientific content.

## Figures and Tables

**Table 1:** General baseline characteristics of the patient cohort.

Characteristic	Number of Patients	Percentage (%)
Total number of patients	96	100%
Sex		
- male	56	58%
- female	40	42%
Age at diagnosis (years)		
- <1 y	3	3%
- 1–9 y	70	73%
- ≥10 y	23	24%
WBC count at diagnosis (×10^9^/L)		
- <20	57	59%
- 20–100	25	26%
- >100	14	15%
Immunophenotypic subtype		
- B-cell ALL	80	84%
- common B-ALL	69	73%
- preB-ALL	7	7%
- proB-ALL	4	4%
- T-cell ALL	12	12%
- proT-ALL	4	4%
- preT-ALL	3	3%
- T cortical-ALL	4	4%
- ETP-ALL	1	1%
- MPAL (B/myeloid)	3	4%
- MPAL (T/myeloid)	1	1%
CNS status		
- CNS 1	86	90%
- CNS 2	2	2%
- CNS 3	3	3%
- CNS NA	5	5%

WBC—white blood cell; CNS—central nervous system; ALL—acute lymphoblastic leukemia; MPAL—mixed phenotype acute leukemia; ETP—early T-cell precursor.

**Table 2:** Treatment response based on FC-MRD.

Timepoint	FC-MRD Response	Number of Patients	Percentage (%)
Day 15	<0.1%	25	26%
	0.1–10%	45	47%
	>10%	22	23%
	NA	4	4%
Day 33	Negative (<5 × 10^−4^)	77	80%
	Positive (≥5 × 10^−4^)	9	10%
	NA	10	10%
Day 78	Negative (<5 × 10^−4^)	79	82%
	Positive (≥5 × 10^−4^)	1	1%
	NA	16	17%

FC-MRD—flow cytometry minimal residual disease; NA—not available.

**Table 3:** Cytogenetic and molecular findings in the study cohort.

Genetic Mutations	Number of Patients	Percentage (%)
Total number of patients	96	100%
Conventional cytogenetic		
No evaluable metaphases	26	28%
Normal karyotype	27	28%
HeH (>50 chromosomes)	16	17%
Complex karyotype (≥3 abnormalities)	11	12%
Trisomy 21/Down syndrome	4	4%
Monosomy 7	2	2%
Monosomy 10	1	1%
Monosomy 18	1	1%
Trisomy 10	1	1%
t (8;14)	1	1%
t (10;14)	1	1%
Other rare cytogenetic abnormalities *	5	5%
FISH		
FISH-negative	84	89%
*KMT2A* rearrangement	4	3%
*TCF3* rearrangement	3	3%
*RUNX1* rearrangement	1	1%
Del(9p)	1	1%
Monosomy 7	1	1%
Monosomy 8	1	1%
RT-PCR (fusion genes)		
Negative	75	78%
*ETV6::RUNX1*	17	17%
*KMT2A::MLLT3*	2	2%
*KMT2A::AFF1*	2	2%
*TCF3::PBX1*	1	1%
*FLT3::ITD*	1	1%

* Includes marker chromosomes, derivative X chromosome [46,X,der(X)]; HeH—high hyperdiploidy; FISH—fluorence in situ hybridization; RT-PCR—real-time PCR.

**Table 4:** Treatment response and survival outcomes according to genetic subtypes in the study cohort.

Genetic Subtypes	Nr. Positive Patients	MRD Day 15 <0.1%	MRD Day 15 0.1–10%	MRD Day 15 ≥10%	MRD TP1 Positive	MRD TP2 Positive	Relapse	2y EFS *p* (Log-Rank)	2y OS *p* (Log-Rank)
*ETV6::RUNX1*	17	7	8	2	0	0	none	94.1% (95% CI: 83–100) (*p* = 0.27)	94.1% (95% CI: 83–100) (*p* = 0.51)
HeH	16	6	6	4	0	0	none	93.4% (95% CI: 82–100) (*p* = 0.44)	93.8% (95% CI: 82–100) (*p* = 0.69)
Del*IKZF1*	7	2	4	1	1	0	medullary (n = 1)	71.4% (95% CI: 38–100) (*p* = 0.24)	71.4% (95% CI: 38–100) (*p* = 0.12)
Del*CDKN2A/CDKN2B*	14	3	3	6	2	1	CNS (n = 1)	85.7% (95% CI: 68–100) (*p* = 0.79)	92.9% (95% CI: 80–100) (*p* = 0.80)
Del*PAX5*	11	3	6	2	1	1	CNS (n = 1)	90% (95% CI: 75–100) (*p* = 0.73)	100% (95% CI: 70–100) (*p* = 0.43)
Del*ETV6*	5	1	1	3	1	1	none	80% (95% CI: 45–100) (*p* = 0.48)	80% (95% CI: 45–100) (*p* = 0.33)
*iAMP21*	4	2	1	1	0	0	none	100% (95% CI: 50–100) (*p* = 0.60)	100% (95% CI: 50–100) (*p* = 0.69)
*KMT2A*	4	2	0	2	1	1	medullary (n = 1)	80% (95% CI: 45–100) (*p* = 0.63)	80% (95% CI: 45–100) (*p* = 0.43)
*KRAS/NRAS*	23	2	12	11	2	0	CNS (n = 1) and medullary (n = 1)	84.6% (95% CI: 71–98) (*p* = 0.65)	88.5% (95% CI: 76–100) (*p* = 0.64)
Ph-like	19	6	12	1	2	0	combined CNS and medullary (n = 1)	84.2% (95% CI: 68–100) (*p* = 0.91)	89.5% (95% CI: 76–100) (*p* = 0.95)
*NOTCH1*	7	1	4	2	0	0	medullary (n = 1) and CNS (n = 1)	71.4% (95% CI: 38–100) (*p* = 0.22)	100% (95% CI: 60–100) (*p* = 0.58)
*CDK4*	12	3	6	3	1	0	none	83.3% (95% CI: 62–100) (*p* = 0.48)	83.3% (95% CI: 62–100) (*p* = 0.49)
*ZNF384*	6	1	1	4	0	0	none	100% (95% CI: 60–100) (*p* = 0.35)	100% (95% CI: 60–100) (*p* = 0.54)

positive MRD defined as ≥5 × 10^−4^ (FC-MRD).

## Data Availability

The original contributions presented in this study are included in the article/Supplementary Materials. Further inquiries can be directed to the corresponding author.

## Supplementary Materials

The following supporting information can be downloaded at: https://www.mdpi.com/article/10.3390/ijms27104517/s1.

## Abbreviations

The following abbreviations are used in this manuscript:

ALL	acute lymphoblastic leukemia
BFM	Berlin–Frankfurt–Münster
CNA	copy number alteration
CNS	central nervous system
EFS	event-free survival
FC	flow cytometry
FISH	fluorescence in situ hybridization
HeH	high hyperdiploidy
HR	high risk
iAMP21	intrachromosomal amplification of chromosome 21
IR	intermediate risk
MLPA	multiplex ligation-dependent probe amplification
MPAL	mixed phenotype acute leukemia
MRD	minimal residual disease
NGS	next-generation sequencing
OS	overall survival
Ph-like	PhilaDelphia chromosome-like
RT-PCR	real-time polymerase chain reaction
SR	standard risk
WBC	white blood cell
WHO	World Health Organization

## Appendix A

### Appendix A.1. Cytogenetics Methods

Bone marrow cells were cultured using standard cytogenetic protocols. Cells were arrested in metaphase with colcemid treatment. GTG banding was performed for karyotype analysis. A minimum of 10 metaphases per sample were analyzed and results were interpreted according to the International System for Human Cytogenomic Nomenclature ISCN 2020 [49]. FISH analysis included KMT2A (MLL) breakapart and BCR::ABL1 dual fusion probes. Additional FISH probes were selected on a case-by-case basis according to cytogenetic findings observed on conventional karyotyping, in order to further characterize suspected structural abnormalities.

### Appendix A.2. Gene Fusions Screening

We performed targeted screening for recurrent gene fusions associated with acute lymphoblastic leukemia (ALL) using RNA extracted from leukemic cells from peripheral blood samples, followed by reverse transcription and amplification through multiplex PCR. The panel included the clinically relevant fusion transcripts: TCF3::PBX1 (t(1;19) (q23;p13)), KMT2A::AFF1 (t(4;11) (q21;q23)), KMT2A::MLLT3, BCR::ABL1 p190 and p210 (t(9;22) (q34;q11)), *ETV6*::*RUNX1* (t(12;21) (p13;q22)), and SIL::TAL1 (del(1) (p32;p32)).

### Appendix A.3. MLPA Analysis

Peripheral blood samples were collected for DNA extraction. The DNA sample was analyzed for Deletions and duplications in the genes *IKZF1* (exons 1–8), CDKN2A (exons 2, 4), CDKN2B (exon 2), *PAX5* (exons 1, 2, 5, 6, 7, 8, 10), *ETV6* (exons 1, 2, 3, 5, 8), *RB1* (exons 6, 14, 19, 24, 26), *EBF1* (exons 1, 10, 14, 16), BTG1 (exons 1, 2), AREA-down, SHOX-AREA-down, CRLF2 (exon 4), CSF2RA (exon 10), IL3RA (exon 1), and P2RY8 (exon 2, RUO), using the SALSA^®^ MLPA Probemix P335 ALL-*IKZF1* kit (MRC Holland, Amsterdam, The Netherlands) and the SeqStudio™ Genetic Analyzer System (Applied Biosystems™, Thermo Fisher Scientific, Waltham, MA, USA). Additionally, the SALSA^®^ MLPA Probemix P327-B2 iAMP21-ERG kit (MRC Holland, RUO) was used to screen for intrachromosomal amplification of chromosome 21 (iAMP21), targeting genes including HSPA13 (exon 5), SAMSN1 (exon 3), MIR99A, BTG3 (exon 3), TMPRSS15 (exon 10), NCAM2 (exon 4), MIR155, APP, CYYR1 (exon 3), ADAMTS5 (exon 4), BACH1 (exon 6), TIAM1 (exon 23), OLIG2 (exon 2), KCNE2 (exon 2), *RUNX1* (exons 2, 4, 6, 7, 8, 9), SIM2 (exon 11), HLCS (exon 7), DYRK1A (exon 3), KCNJ6 (exon 4), ERG (exons 1–10), ETS2 (exon 9), PSMG1 (exon 6), TMPRSS2 (exon 6), RIPK4 (exon 2), TFF1 (exon 3), ITGB2 (exon 7), SLC19A1 (exon 7), COL6A2 (exon 2), and PRMT2 (exon 4). MLPA was performed in accordance with the manufacturer’s instructions.

The relative copy number was determined by comparing the normalized peak heights of target-specific probes and reference probes in each test sample with those from control samples with a known normal copy number profile. Analysis was performed using Coffalyser.Net software^TM^ version 240429.1451 (MRS Holland, Amsterdam, The Netherlands). For interpretation, we considered the median ratio values of overexpressed probes (marked in blue by the software). To avoid false-positive results, particular attention was given to patients with cytogenetic abnormalities such as trisomy 21, hyperdiploidy or complex karyotypes. Heterozygous duplications typically presented with a median ratio of approximately 1.6, while homozygous duplications showed a median above 1.8. Samples were considered to have a normal copy number when the median ratio was below 1.3. In such cases, additional verification was performed using fluorescence in situ hybridization (FISH) with *RUNX1* probes to assess the number of *RUNX1* signals. A count of more than five *RUNX1* signals per nucleus was considered indicative of iAMP21, while fewer than five signals excluded this amplification.

### Appendix A.4. Targeted NGS

Targeted NGS was performed using the Illumina TruSight™ Oncology 500 (TSO500) hybrid-capture-based assay, which is designed to detect multiple classes of mutations: single-nucleotide variants, multi-nucleotide variants (<3 bp), small Insertions (1–18 bp)/Deletions (1–27 bp) and Copy Number Variants (CNVs). The assay also detects, quantitatively, microsatellite instability (MSI) and tumor mutational burden (TMB). Fusions and splice variants are detected in RNA. DNA and RNA are extracted from the same FFPE tissue using the Allprep DNA/RNA FFPE Kit (Qiagen, Inc., Hilden, Germany). RNA is then reverse transcribed to cDNA. The genomic DNA and cDNA are sheared to prepare sequencing libraries. The regions of interest are hybridized to biotinylated probes, magnetically pulled down with streptavidin-coated beads, and eluted to enrich the library pool. Finally, libraries are normalized using a simple bead-based protocol, then pooled and sequenced on an Illumina NextSeq 500 instrument (Illumina, San Diego, CA, USA).

Variant classification was performed in accordance with the joint consensus recommendations of the Association for Molecular Pathology, the American Society of Clinical Oncology and the College of American Pathologists [50]. According to this framework, variants are categorized into Tiers IA, IB, IIC, IID, III, and IV, reflecting descending levels of clinical significance for the patient, in compliance with the consensus guidelines. For this study, only variants classified as Tier IA, IB and IIC were included in the analysis. Tier IA variants represent alterations of strong clinical significance supported by Level A evidence, corresponding to FDA-approved therapies or established clinical practice guidelines in the relevant tumor type, whereas Tier IB variants indicate strong clinical significance supported by Level B evidence derived from well-powered studies or expert consensus in the same disease context. Tier IIC variants include alterations with potential clinical significance supported by emerging or limited clinical evidence, typically extrapolated from other tumor types or early-phase clinical studies.

Variant annotation and assignment of clinical significance were supported by curated reference databases integrated within the bioinformatic pipeline, including genomic build GRCh37.p13; NCBI RefSeq v105; ClinVar (20240603); COSMIC v100; dbNSFP v4.4c; dbSNP build 149; ExAC v1.0; NHLBI Exome Sequencing Project v0.0.30; and gnomAD release r2.1.
