# Supplementary material for: Integrated Genomic Profiling of Pediatric Acute Lymphoblastic Leukemia: Genomic Landscape, Risk Stratification and Association of RAS Pathway Mutations with Early Treatment Response
Source: Int J Mol Sci. 2026 May 18;27(10):4517. doi: 10.3390/ijms27104517 (PMC13207613; doi:10.3390/ijms27104517)
Supplement: Supplementary file 1 [file ijms-27-04517-s001.zip › ijms-4310461-supplementary.pdf]

Supplementary material

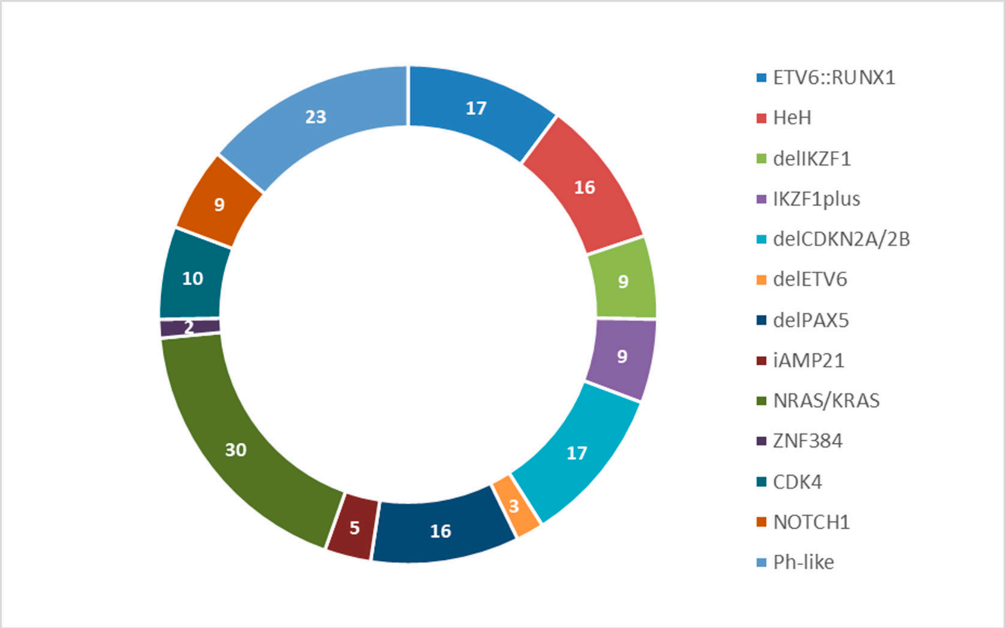

Figure S1. Distribution of recurrent genomic alterations within the intermediate-risk (IR) subgroup. Donut chart illustrating the distribution (percentage) of recurrent genomic alterations identified in patients classified as IR according to the ALLIC BFM 2022 protocol. KRAS/NRAS alterations represented the most frequent findings, followed by CDKN2A/CDKN2B deletions and Ph-like alterations.
